# Peer Review of “Exercise-Induced Hypoalgesia Following Proprioceptive Neuromuscular Facilitation and Resistance Training Among Individuals With Shoulder Myofascial Pain: Randomized Controlled Trial”

**DOI:** 10.2196/45060

**Published:** 2022-12-27

**Keywords:** exercise induced hypoalgesia, proprioceptive neuromuscular facilitation, PNF, resistance exercise, conditioned pain modulation, myofascial pain syndrome, resistance training, hypoalgesia, exercise-induced hypoalgesia, shoulder myofascial pain, myofascial pain, pain management, chronic pain, musculoskeletal pain, physical therapy, physiotherapy, shoulder pain, upper back pain, exercise, pain

*This is a peer-review report submitted for the paper “Exercise-Induced Hypoalgesia Following Proprioceptive Neuromuscular Facilitation and Resistance Training Among Individuals With Shoulder Myofascial Pain: Randomized Controlled Trial”*

## Round 1 Review

### General Comments

This paper [1] aims to compare short-term exercise-induced hypoalgesia responses following different types of exercise in pain modulation within patients with myofascial pain. It is generally well written and presents innovative results to clarify the knowledge in the treatment of myofascial pain.

### Specific Comments

#### Major Comments

1. Methods: In the procedures section, please add information about possible blinding of the evaluators (ie, experience of the persons who did the manual assessment of the myofascial pain syndrome, people who performed the exercise programs, etc).

#### Minor Comments

2. Discussion: Please try to address the important improvements in the proprioceptive neuromuscular facilitation exercise group in relation to personal interaction with the researcher (manual contact, personal adaptation, etc).
